# What’s new in musculoskeletal oncology

**DOI:** 10.1186/s12891-021-04590-1

**Published:** 2021-08-17

**Authors:** Costantino Errani, Andreas F. Mavrogenis, Shinji Tsukamoto

**Affiliations:** 1 Department of Orthopaedic Oncology, IRCCS Istituto Ortopedico Rizzoli, Via Pupilli 1, 40136 Bologna, Italy; 2 First Department of Orthopedics, School of Medicine, National and Kapodistrian University of Athens, 41 Ventouri Street Holargos, 15562 Athens, Greece; 3 Department of Orthopaedic Surgery, Nara Medical University, 840, Shijo-cho, Nara 634-8521 Kashihara-city, Japan

**Keywords:** Musculoskeletal tumors, Bone tumors, Soft tissue tumors, Soft tissue sarcomas, Bone sarcomas, Metastatic bone tumor, Bone metastases, What’s new, New treatments

## Abstract

We reviewed the recent literature related to primary musculoskeletal tumors and metastatic bone tumors. With regard to primary bone tumors, computer navigation systems and three-dimensional-printed prostheses seem to be new treatment options, especially in challenging anatomical locations, such as the sacrum and pelvis. Regarding the treatment of giant cell tumor of bone, recent studies have suggested that denosumab administration is related to a higher local recurrence rate following curettage, but a lower local recurrence rate following en bloc resection. In addition, there was no difference in the local recurrence rate at five years after surgery between short-term and long-term denosumab therapy. With regard to soft tissue tumors, percutaneous cryoablation appears to be a new treatment option for extra-abdominal desmoid tumors, with encouraging results. Regarding soft tissue sarcomas, a negative surgical margin of < 1 mm is sufficient to control local recurrence. Pexidartinib seems to be a promising systemic therapy for the treatment of tenosynovial giant cell tumors for which surgery is not expected to improve the function of the affected limb. Finally, the life expectancy of patients is the most important factor in determining the optimal surgical procedure for patients with impending or pathological fractures of the long bone due to metastatic bone tumors. Elevated C-reactive protein level was found to be an independent poor prognostic factor at 1 year after surgery for long bone metastases.

## Background

This editorial will highlight the recent developments in the treatment of benign and malignant musculoskeletal tumors over the last few years. We reviewed the literature to determine the newer aspects of the treatment of primary musculoskeletal tumors and metastatic bone tumors. With regard to primary bone tumors, computer navigation systems appear to be useful in the resection of tumors in areas with complex local anatomy, such as the sacrum and pelvis. In addition, the use of custom-made three-dimensional (3D)-printed prostheses for limb salvage surgery in bone tumors seems to be a new treatment option. Finally, many researchers have endeavored to find new potential targets for systematic therapy not only in malignant tumors but also in benign tumors, such as giant cell tumor of bone (GCTB) and diffuse tenosynovial giant cell tumor (TGCT). Considering the risk of surgical complications, life expectancy appears to be crucial in deciding the ideal treatment option for patients with bone metastases. Several authors have created models to predict the prognosis of patients with metastatic bone disease.

## Main text

### Bone tumors

**Fig. 1:**
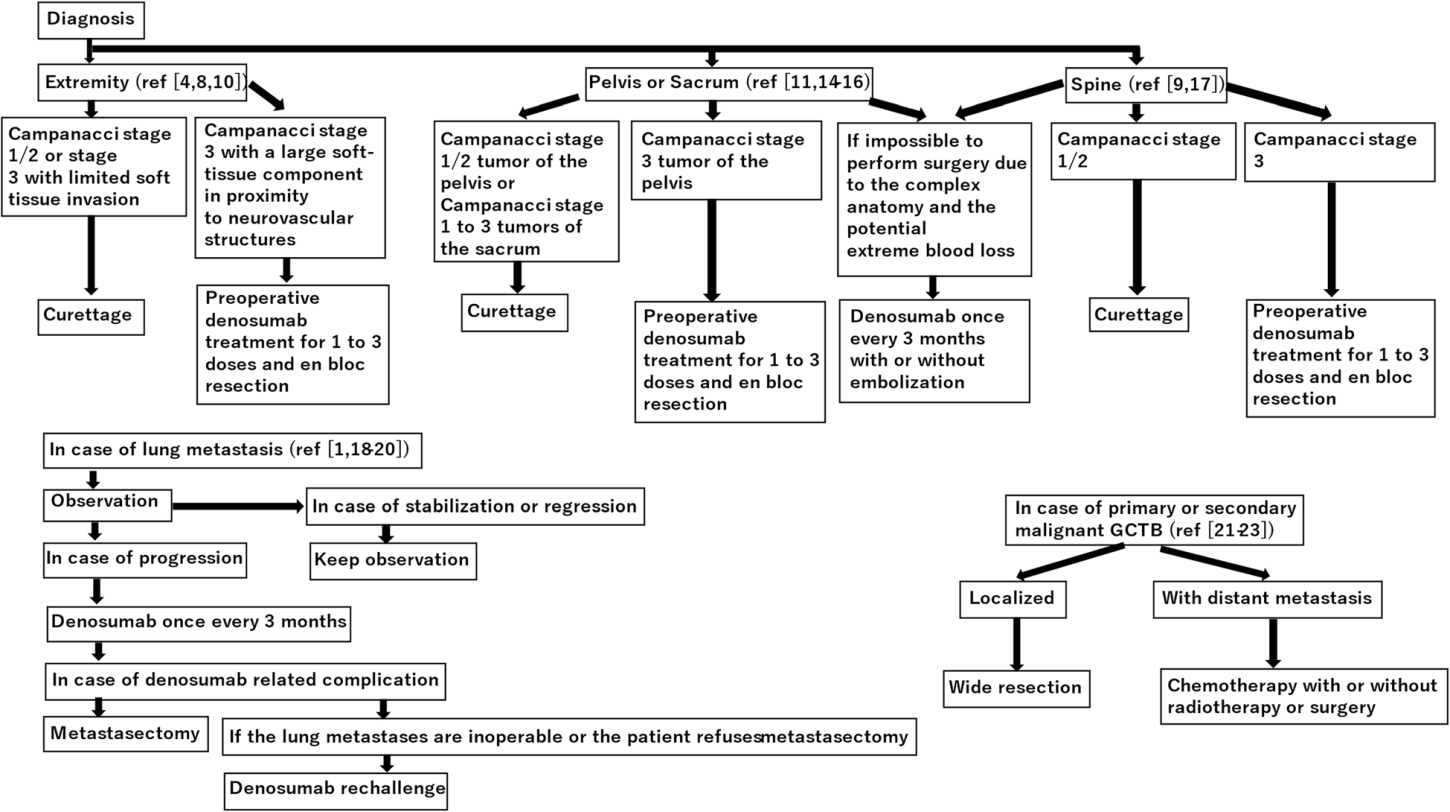
The treatment algorithm for giant cell tumor of bone. GCTB: giant cell tumor of bone

The ideal treatment for GCTB remains controversial. In 2013, the U.S. Food and Drug Administration approved the use of denosumab in patients with unresectable GCTB or those with GCTB who are expected to have severe surgical morbidity [1]. Chawla et al. reported that the disease control rate of denosumab therapy for inoperable or metastatic GCTB is up to 96 % [1]. However, because complications (such as hypophosphatemia, osteonecrosis of the jaw, peripheral neuropathy, skin rash, and atypical femoral fracture) associated with long-term administration of denosumab have been reported [2], a clinical trial (denosumab 120 mg every 3 months) with reduced dose density to reduce these complications is ongoing in patients with unresectable GCTB (REDUCE study)(NCT03620149). In 2015, Rutkowski et al. reported that for patients with resectable GCTB, preoperative denosumab therapy resulted in beneficial surgical downstaging [3]. According to a recent systematic review, the recurrence rate was 20–100 % in the group that received preoperative denosumab therapy and curettage, while it was 0–50 % in the group that received curettage alone [4]. This is because preoperative administration of denosumab causes osteosclerosis, which makes it difficult to identify the tumor area intraoperatively, leaving the tumor behind, and the tumor cells hidden in the osteosclerotic lesion are reactivated after the denosumab is discontinued [4–7]. Medellin et al. reported a local recurrence rate of 24 % (11 of 45 patients) in the en bloc resection alone group, whereas it was 0 % (none of 3 patients) in the en bloc resection combined with preoperative denosumab therapy group [8]. It has been suggested that denosumab administration before en bloc resection may harden the tumor, reduce tumor spillage, and reduce the risk of local recurrence [9]. Hindiskere et al. analyzed 161 patients with GCTB who received preoperative denosumab treatment for downstaging prior to surgery. They reported that there was no difference between short-term and long-term administration of denosumab in terms of radiologically objective tumor response (90 % [43 of 48] vs. 81 % [29 of 36]; *p* = 0.24), histological response (79 % [38 of 48] vs. 83 % [30 of 36]; *p* = 0.81), and local recurrence-free survival rate at five years after surgery (73 % vs. 64 %; *p* = 0.50) [10]. They suggested that reducing the preoperative dose of denosumab can reduce the complications and costs of denosumab [10]. These studies suggest that physicians should carefully consider the use of denosumab before curettage of GCTB, and confirm that a short course of denosumab before resection can make surgery easier, with a lower risk of local recurrence. Guo et al. retrospectively investigated 27 patients with GCTB around the acetabulum who underwent surgery [11]. Local recurrence was observed in 4 of 13 patients (31 %) who had Campanacci stage 2 tumors and underwent curettage. Local recurrence was observed in none of 14 patients (0 %) who had Campanacci stage 3 tumors and underwent en bloc resection [11]. The mean Musculoskeletal Tumor Society (MSTS) score was 24 in the 13 patients who underwent curettage and 22 in the 14 patients who underwent en bloc resection. Thrombosis (1 patient) and infection (1 patient) occurred in 2 patients who underwent curettage, while infection (5 patients), nonunion (1 patient), and dislocation (1 patient) occurred in 7 patients who underwent en bloc resection [11]. Most sacral GCTBs occur at levels including S1-2 [12], and wide resection, including the nerve roots of S1-3, can reduce the local recurrence rate, but it causes severe functional loss, such as motor deficits and bowel, bladder, or sexual dysfunction, as well as lumbopelvic discontinuity [13]. Therefore, wide resection is usually unacceptable for the treatment of benign bone tumors [13]. Zhao et al. reported that the local recurrence rate of sacral GCTB after nerve-sparing surgery (curettage) was 29 % (33/114 patients) [14]. In 26 patients with sacral or pelvic GCTBs, Sambri et al. reported a local recurrence rate of 0 % (none of 3 patients) in the en bloc resection combined with pre- and post-operative denosumab treatment group, while it was 62 % (5 of 8 patients) in the curettage combined with pre- and post-operative denosumab treatment group [15]. Puri et al. investigated the clinical outcomes of 13 patients with sacral GCTB treated non-surgically [16]. Non-surgical treatments include various combinations of short-term denosumab, embolization, and radiotherapy [16]. No further treatment was performed if the lesions stopped growing [16]. If the lesion grew, additional denosumab and/or embolization were performed until the tumor was locally controlled [16]. As a result, 10 patients (77 %) had no progression and remained asymptomatic, 2 patients (15 %) remained with occasional pain (but with stable disease), and 1 patient (8 %) died due to other causes [16]. The average total number of denosumab doses was 9 (range, 5–16), and the average total number of embolizations was 4 (range, 0–12) [16]. Eight patients underwent radiotherapy. Patients with bladder dysfunction recovered [16]. Boriani et al. retrospectively investigated 49 patients with spinal GCTB who underwent surgery. Among the 24 patients with Enneking stage 3 tumors, 8 of 13 patients who underwent curettage and 1 of 11 patients who underwent en bloc resection had local recurrence. Among the 18 patients with Enneking stage 2 tumors, 1 of 16 patients who underwent curettage and none of the 2 patients who underwent en bloc resection had local recurrence [17]. Boriani et al. reported no recurrence in 4 patients with spinal GCTB who underwent preoperative denosumab treatment and en bloc resection [9]. Therefore, denosumab administration before curettage may not be recommended for GCTBs of the pelvis, sacrum, and spine. In contrast, short-term denosumab administration prior to en bloc resection may be recommended. Non-surgical treatments, such as denosumab treatment and embolization, are treatment options for large tumors with a high risk of massive bleeding and nerve injury. Tsukamoto et al. reported that 45 % (10/22) of patients with lung metastases who were initially managed with the wait-and-see approach had stable disease [18]. They reported that 36 % of the 22 patients required metastasectomy, and 9 % required treatment with denosumab [18]. Therefore, it may be recommended to observe lung metastases secondary to GCTB as a first-line approach. Even if discontinuation of denosumab is required due to denosumab-related complications, it has been reported that resumption of denosumab treatment is possible and effective [18–20]. With regard to the treatment of localized malignant GCTB, Liu et al. [21] reported that adjuvant chemotherapy did not prolong overall survival. Conversely, lung metastasis-free survival benefit was observed with adjuvant chemotherapy. The 5-year survival rates in the chemotherapy and no-chemotherapy groups were 57 and 33 %, respectively (*p* = 0.167) [21]. The median pulmonary metastasis-free survival in patients who underwent adjuvant chemotherapy was significantly longer than that in patients who underwent surgery alone (13 months vs. 6 months) [21]. Therefore, wide resection may be recommended for localized malignant GCTBs. Palliative chemotherapy, radiotherapy, and surgery are recommended for malignant GCTBs with distant metastases [21–23]. Following these studies, we suggest a treatment algorithm for GCTB (Fig. 1).

Various treatment options for solitary bone cysts have been reported to date, including allograft or autograft bone grafting following curettage, injection of autogenous bone marrow or steroids, and insertion of an elastic stable intramedullary nail. In a retrospective study, Zhang et al. reported that the complete cure rate according to the Capanna criteria of autogenous bone marrow injection combined with an elastic stable intramedullary nail, elastic stable intramedullary nail alone, and autogenous bone marrow injection alone were 96 %, 82 %, and 46 %, respectively [24]. Zhang et al. reported that the complete cure rate of steroid injection combined with an elastic stable intramedullary nail was 78 % [25]. Therefore, the use of elastic stable intramedullary nails seems to be a viable and highly effective treatment option for solitary bone cysts.

There is no evidence that enchondromas and atypical cartilaginous tumors (ACT) should be treated surgically. Omlor et al. retrospectively studied 228 patients with enchondromas or ACTs of the extremities treated surgically with curettage (75 patients) and conservatively (153 patients) [26]. In the surgery group, seven patients had complications, 86 % of which were osteosynthesis related, and three patients had local recurrence. In a group of patients who were managed conservatively, imaging over time showed no significant changes in the lesions. Both the surgery and conservative groups had excellent MSTS scores (96 % vs. 97 %, respectively) [26]. The conservative approach with careful clinical and radiographic follow-up seems to be safe for enchondromas and ACTs. Gulia et al. analyzed 427 patients with chondrosarcomas, including 53 patients with grade 1 chondrosarcoma, 330 patients with grade 2 chondrosarcoma, and 41 patients with grade 3 chondrosarcoma. They reported that 3 out of 330 patients with grade 2 chondrosarcoma had bone metastases [27]. Patients with grade 1 or grade 3 chondrosarcomas did not have bone metastases. They suggested that bone images should be excluded from traditional chondrosarcoma staging and surveillance [27].

Thévenin-Lemoine et al. investigated whether pre- or post-chemotherapy magnetic resonance imaging (MRI) provides more accurate tumor limits in determining the extent of Ewing’s sarcoma resection [28]. They found that post-chemotherapy MRI was able to delineate the tumor limits more accurately than pre-chemotherapy MRI. The median difference between pre-chemotherapy T1 MRI and histological measurements was 19 mm, whereas that between post-chemotherapy T1 MRI and histological measurements was 5 mm. Adding a margin of 20 mm or more to the tumor limit on T1 MRI after chemotherapy always provides a safe histological margin [28].

Andreou et al. analyzed 180 patients with localized Ewing sarcoma of the pelvis and sacrum [29]. Sacral tumors had a lower local recurrence rate (12 % vs. 28 % at 5 years, *p* = 0.032), higher event-free survival rate (66 % vs. 50 % at 5 years, *p* = 0.026), and higher overall survival rate (72 % vs. 56 % at 5 years, *p* = 0.025) than pelvic tumors [29]. Between patients with sacral tumors who received definitive radiotherapy and those who received surgery and adjuvant radiotherapy, there was no difference in the rate of local recurrence (17 % vs. 0 % at 5 years, *p* = 0.125) or overall survival (73 % vs. 78 % at 5 years, *p* = 0.764) [29]. In pelvic tumors, the surgery and adjuvant radiotherapy group had a lower local recurrence rate (14 % vs. 33 % at 5 years, *p* = 0.015) and a higher overall survival rate (72 % vs. 47 % at 5 years, *p* = 0.024) than the surgery alone group [29]. This study confirmed the important role of radiotherapy in the treatment of Ewing sarcoma of the sacrum and pelvis.

Few studies have investigated whether the interval from the diagnosis of primary malignant bone tumors to the start of treatment affects prognosis. Lawrenz et al. retrospectively studied 2.122 patients with high-grade bone sarcomas included in the National Cancer Database [30]. Time to treatment initiation (TTI) was defined as the number of days from diagnosis to the start of treatment. A 10-day increase in TTI did not reduce overall survival (*p* = 0.72) [30]. This study showed that TTI up to 150 days was not related to the overall survival of localized primary malignant bone tumors. This is useful information for patients delaying treatment in order to receive a second opinion in a higher volume sarcoma center.

Several studies have investigated limb salvage surgery in pediatric patients [31, 32]. A recent study retrospectively investigated 46 children with femoral sarcomas who underwent intercalary resection and biological reconstruction [32]. Twenty-five patients were reconstructed with an allograft combined with a vascularized free fibula, and 21 patients were reconstructed with allografts alone. There was no difference in reconstruction survival between the allograft combined with vascularized free fibula group and the allograft alone group (84 % vs. 87 %, respectively; *p* = 0.89) [32]. Because the addition of vascularized free fibula is more time consuming and costly and does not appear to contribute to improving bone union rates or reducing complications, femoral intercalary defects can be reconstructed with allografts only, and a vascularized fibula autograft can be used to rescue allografts only in the event of an allograft fracture or nonunion.

Tsuda et al. retrospectively investigated 124 skeletally immature children reconstructed with extendable endoprosthetic replacement [33]. The 10-year prosthesis failure-free survival rate was 28 %, and 90 % of the patients had a total of 243 complications. Of these, soft tissue-related complications were the most common (27 %). Soft-tissue failures were mostly common in the proximal femur (77 %; *p* = 0.003), and aseptic loosening (52 %; *p* = 0.014) and structural failure (55 %; *p* = 0.001) occurred most frequently in the distal femur. Excluding lengthening surgery, 105 patients (85 %) underwent additional surgery, with an average of 2.7 surgeries per patient. The affected limb was salvaged in 113 patients (91 %) [33].

Gupta et al. retrospectively reviewed 32 patients who underwent iliac wing or iliosacral resection without reconstruction for primary bone tumors [34]. Eighteen complications occurred in 17 patients: 13 wound healing deficiencies/infections, three fractures, one hip dislocation, and one pulmonary embolism. The mean MSTS score was 67 %. These patients had good limb function, even without bony reconstruction. They suggested leaving the frailty of the segment instead of reconstructing the defect. Over time, iliac sacral arthrodesis or pseudarthrosis may develop. This surgical method minimizes complications and improves the functional results [34]. Jin et al. evaluated the functional results of 21 patients following iliosacral resection: 18 patients had no reconstruction (group 1) and three patients underwent reconstruction with autografts (group 2) [35]. The limb-length discrepancy was similar between groups 1 and 2 (mean 1.7 vs. 1.0 cm, respectively). The average MSTS scores for groups 1 and 2 were 94 and 93 %, respectively. The mean Toronto Extremity Salvage Scores between groups 1 and 2 were similar (98 and 98.5, respectively) [35]. These studies seem to recommend going back and not reconstructing skeletal defects of the pelvis or sacrum to minimize complications. However, Ji et al. reported early results of 80 patients who underwent three-dimensional (3D)-printed modular hemipelvic prosthetic reconstruction after resection of periacetabular tumors [36]. The mean MSTS score was 84 %. Sixteen patients (20 %) experienced complications: eight patients (10 %) experienced wound dehiscence, five patients (6 %) experienced deep infections, and two patients (3 %) experienced dislocations. None of the patients experienced aseptic loosening. This prosthesis showed stable fixation and acceptable initial functional outcomes [36]. Recent advances in 3D printing technology and prosthesis porosity creation in the manufacture of custom materials have the potential to improve implant incorporation and the durable lifespan, reducing the risk of complications related to this demanding surgery.

Navigation is a useful adjunct in the resection of malignant tumors located in areas such as the sacrum and pelvis, which are difficult to resect with adequate margins. Bosma et al. compared the outcomes of 36 patients who underwent resection of sacral or pelvic sarcomas using intraoperative navigation in 34 patients who underwent resection without navigation [37]. The proportion of the patients who underwent resection using navigation and achieved adequate margins was higher than that of the patients who did not (29 of 36 patients [81 %] vs. 17 of 34 [50 %]; *p* = 0.007). There was no difference in soft-tissue margins between the navigation and no-navigation groups (18 of 36 patients [50 %] vs. 18 of 34 [54 %]; *p* = 0.995). Intraoperative navigation guidance facilitates resection with negative bone margins but not soft tissue margins in surgical resections of sacral and pelvic sarcomas [37]. Computer-assisted technology to assist in tumor resection requires further progress, but this study showed that this technology can be a useful aid in complex sacral or pelvic resection.

Sanders et al. retrospectively analyzed 18 patients who had infections after resection of a tumor around the acetabulum and reconstruction with a prosthesis. Fourteen (78 %) of them developed polymicrobial infections. Enterobacteriaceae were detected in 67 % of them (12 of 18). Microorganisms associated with the intestinal flora were identified in 76 % of the cases. The polymicrobial flora found in the series may justify the use of broader spectrum antibiotic prophylaxis aimed at gram-negative bacteria in pelvic reconstruction following bone tumor resection [38].

In summary of the bone tumors section, regarding the treatment of GCTB, recent studies have suggested that denosumab is related to a higher local recurrence rate following curettage but a lower local recurrence rate following en bloc resection. In addition, there was no difference in the local recurrence rate at five years after surgery between short-term and long-term denosumab therapy. Computer navigation systems and 3D-printed prostheses seem to be new treatment options, especially in challenging anatomical locations, such as the sacrum and pelvis.

### Soft tissue tumors

Hirai et al. analyzed 139 patients who had schwannomas occurring in major nerves and reported the incidence of neurological complications after surgical treatment [39]. This multi-institute retrospective study aimed to identify the factors that predict the occurrence of complications after enucleation of schwannomas. Forty-nine patients (35 %) had postoperative complications. Forty-two patients (30 %) had sensory disturbances, such as numbness, pain, and hypoesthesia. Eight patients (6 %) developed motor weakness. Multivariate analysis showed that the tumors originating from the major motor nerves had a higher incidence of postoperative complications (*p* = 0.03). Sensory complications gradually improved in 41 of 42 patients, but 6 of 8 patients with motor weakness did not recover to preoperative levels [39]. The results of this study help clinicians discuss the risk of postoperative complications in patients with schwannomas.

**Fig. 2:**
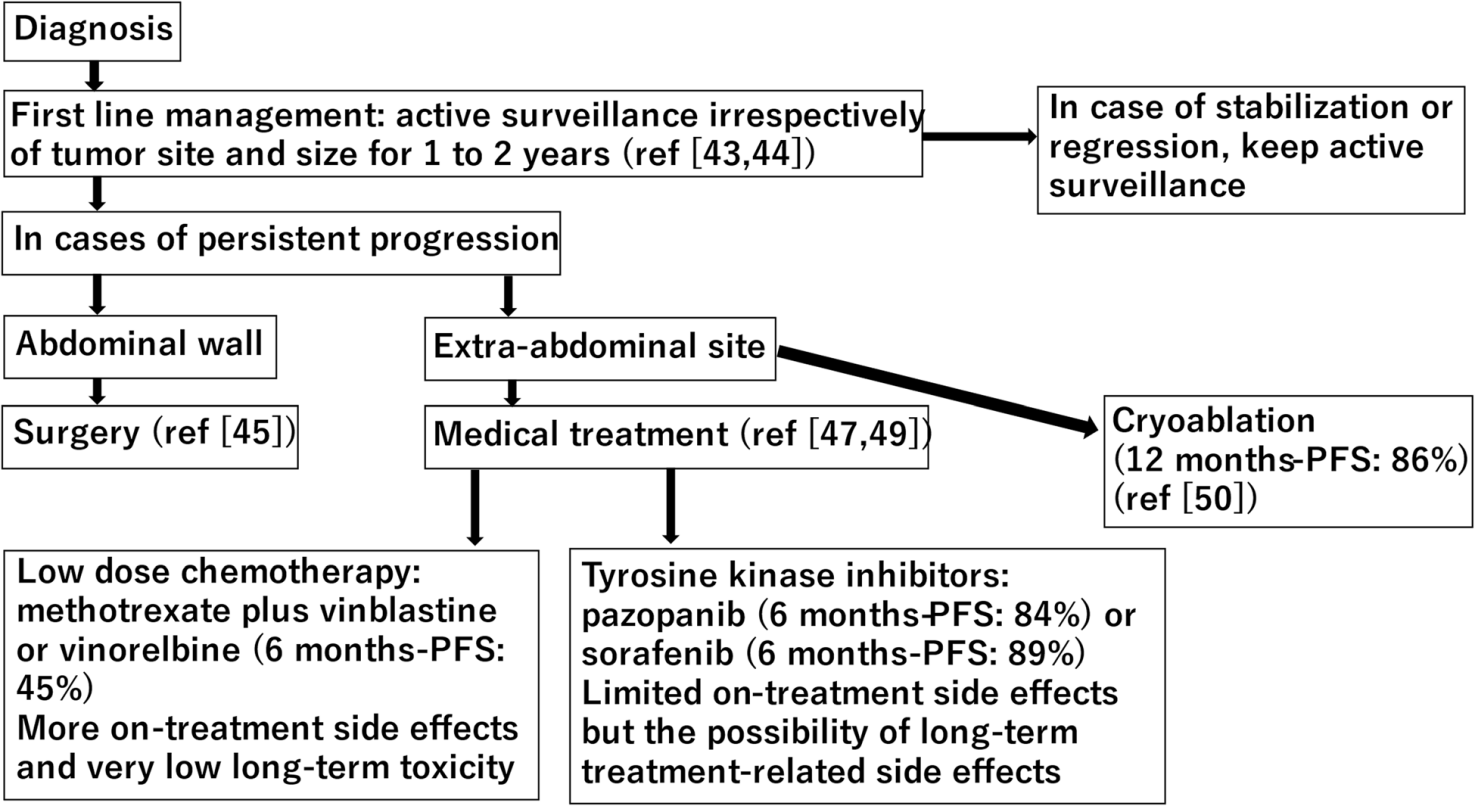
The treatment algorithm for desmoid tumors. PFS: progression-free survival

A recent study retrospectively investigated the outcomes of 99 patients with extra-abdominal desmoid tumors [40]. Sixty patients received local treatment (45 patients underwent surgery only and 15 patients underwent surgery and adjuvant radiotherapy), and 39 patients underwent observation (16 patients) or medical treatment (16 patients received low-dose chemotherapy and 7 patients received non-steroidal anti-inflammatory drugs [NSAIDs] alone or a combination of NSAIDs and anti-hormonal therapy [tamoxifen]). In patients who received local treatment, local recurrence was not associated with the surgical margin (*p* = 0.976) or adjuvant radiotherapy (*p* = 0.110). Event-free survival was similar between patients that were observed or received medical treatment as first-line treatment and patients treated with surgery as a first-line treatment (*p* = 0.509) [40]. This study confirms that the role of surgery in the treatment of desmoid tumors is limited and that observation alone is a viable option for desmoid tumors [41]. Newman et al. reported that patients who underwent two or more surgeries and those who underwent surgery and radiotherapy had the lowest Patient-reported Outcomes Measurement Information System function scores [42]. The Desmoid Tumor Working Group recommends the management of asymptomatic patients by first-line observation, regardless of the tumor size and site [43]. If the tumor grows or the symptoms worsen, the decision to start treatment should be made after at least three imaging evaluations over a year or more [44]. Improta et al. stated that the abdominal wall was less dysfunctional after tumor excision, and that surgery should be considered in the event of documented progression of the tumor into the abdominal wall due to the low risk of local recurrence compared to other sites [45]. Sorafenib is an orally active multi-kinase inhibitor that targets mitogen-activated protein kinase, platelet-derived growth factor receptors (PDGFR), vascular endothelial growth factor receptors, and KIT [46]. The results of a double-blind, phase 3 trial that included 87 patients with symptomatic, progressive, or recurrent desmoid tumors, who were randomly assigned to receive either sorafenib (400 mg once daily) or placebo, were reported [47]. After a median follow-up of 27 months, the progression-free survival rate at 2 years was 81 % in the sorafenib group and 36 % in the placebo group (*p* < 0.001) [47]. Pazopanib is a multitargeted tyrosine kinase inhibitor of cKIT, PDGFR, and PDGFRβ [48]. The results of a non-comparative, randomized, open-label, phase 2 trial (DESMOPAZ) by the French Sarcoma Group have been reported [49]. Participants were randomly assigned to receive pazopanib (800 mg/day) or a regimen of vinblastine (5 mg/m²) in combination with methotrexate (30 mg/m²) weekly for 6 months and then every other week for the next 6 months. The proportion of patients treated with pazopanib who had not progressed at 6 months was higher than that of patients treated with methotrexate-vinblastine (84 % vs. 45 %, respectively) [49]: 23 % of the patients in the pazopanib group experienced at least one serious adverse event, as did 27 % of the patients in the methotrexate-vinblastine group [49]. Low-dose chemotherapy has a high disease control rate with a long duration, requires intravenous administration, and has side effects during treatment, but it has no long-term toxicity [44]. Pazopanib and sorafenib are currently attracting attention because they are taken orally and have fewer side effects during treatment and a higher tumor response rate [44]. However, they can cause permanent hypertension or hypothyroidism [44]. This is not a negligible issue because the population of patients is generally young at presentation, with a normal life expectancy [44]. In addition, the long-term safety of the continuous administration of these treatments has never been assessed because these drugs were developed for patients with metastatic cancer, who have a much more limited life expectancy, and, therefore, have less time to develop or show any possible long-term treatment-related effects [44]. Interestingly, a recent multicenter prospective study showed the possible role of cryotherapy in the treatment of desmoid tumors. In a benign tumor in which only chemotherapy seems to be effective, the possibility of treating this tumor with a minimally invasive method, such as cryotherapy, is an interesting novelty [50]. Kurtz et al. analyzed the use of cryotherapy in 50 patients with non-abdominopelvic progressing desmoid tumors. The progression-free survival rate at 12 months was 86 % (complete response, 29 %; partial response, 6 %; stabilization, 31 %, with an objective response rate of 55 %) according to the modified response evaluation criteria for solid tumors. Cryotherapy improved functional outcomes and relieved pain. The treatment results of cryotherapy are poor for large tumors [50]. Following these studies, we suggest a treatment algorithm for desmoid tumors (Fig. 2).

The standard treatment for soft tissue sarcoma is resection with wide margins; however, the relationship between surgical margins and local recurrence remains controversial. Gundle et al. compared the ability of margin classification systems to predict local recurrence rates after soft tissue sarcoma resection [51]. The study group consisted of 2,217 patients with soft tissue sarcomas located in the chest and abdominal wall, extremities, or paraspinal region treated with surgical resection. Surgery alone was performed when at least 1 cm of the normal tissue or fascia was going to be removed with the tumor. Surgery alone could be performed if important structures, such as nerves and blood vessels, were not going to be sacrificed. Otherwise, adjuvant radiotherapy was administered. Margins were assessed by residual tumor (R) classification (in which the microscopic tumor at the inked margin defines R1), the R + 1 mm classification (in which the microscopic tumor within 1 mm of the inked margin defines R1), and the Toronto Margin Context Classification. This study found that the R classification best predicted the risk of local recurrence. After 10 years of follow-up, the local recurrence rates were 8 %, 21 %, and 44 % for R0, R1, and R2, respectively. The R + 1 mm classification increased the R1 margins (726 vs. 278, *p* = 0.001), but led to decreased local recurrence for R1 margins without changing R0 local recurrence, suggesting that a negative margin of < 1 mm may be adequate for the surgical treatment of soft tissue sarcomas [51].

While the efficacy of radiotherapy in combination with surgery is well known in the treatment of soft tissue sarcomas, the role of chemotherapy remains controversial. Callegaro et al. analyzed 3752 patients with soft tissue sarcomas of the extremities [52]. Radiotherapy did not increase the survival rate, but increased the local control rate, especially in myxoid liposarcoma, vascular sarcoma, and myxofibrosarcoma. Chemotherapy did not prolong overall survival (*p* = 0.73) [52]. The effect of chemotherapy for soft-tissue sarcomas remains unclear, and prospective multicenter studies are required to clarify this effect.

Featherall et al. examined the correlation between TTI and overall survival in 8648 patients with localized high-grade soft tissue sarcomas using the National Cancer Database. In the multivariate analysis, delays in treatment of ≥ 42 days tended to shorten the overall survival. This information is useful for discussing delays in starting treatment due to referrals to second opinions and high-volume centers [53].

In summary of the soft tissue tumors section, percutaneous cryoablation appears to be a new treatment option for extra-abdominal desmoid tumors with encouraging results. A negative surgical margin of < 1 mm seems to be sufficient to control local recurrence in the multidisciplinary treatment of soft tissue sarcomas.

### Bone metastases

The life expectancy of patients is the most important factor in determining the optimal surgical procedure for patients with metastatic bone tumors. Therefore, several studies have investigated the prognostic factors that affect the survival of patients with metastatic bone tumors.

Pereira et al. described and identified the factors related to complications within 30 days of index surgery for metastatic disease in the spine [54]. Complications within 30 days occurred in 205 (32 %) of 647 patients. The following variables were correlated with the incidence of complications within 30 days: lower albumin levels (*p* = 0.021), additional comorbidities (*p* = 0.048), pathological fractures (*p* = 0.031), three or more spine levels operated on (*p* = 0.027), and a combined surgical approach (*p* = 0.036). Complications within 30 days after surgery were associated with worse survival (*p* < 0.001) [54]. Bindels et al. retrospectively investigated 1090 patients with long bone metastases who underwent surgery [55]. Postoperative complications occurred within 30 days in 31 % of the patients (333 of 1090). The following factors were related to the occurrence of complications within 30 days: rapidly growing primary tumors classified according to the modified Katagiri classification (*p* = 0.011), pathological fractures (*p* = 0.010), multiple bone metastases (*p* = 0.008), lower extremity bone metastases (*p* < 0.001), hyponatremia (*p* = 0.044), hypoalbuminemia (*p* = 0.002), and elevated white blood cell count (*p* = 0.007). Postoperative complications within 30 days increased mortality within 1 year (*p* < 0.001) [55]. Patients with a high risk of postoperative complications have worse prognoses and should be considered for less invasive treatments.

Ruatta et al. retrospectively reviewed 300 patients with metastatic bone tumors from renal cell carcinoma: 64 patients (21 %) had bone as the only site of metastasis, 22 patients (7 %) had solitary bone metastasis, and 236 patients (79 %) had visceral metastases [56]. The median overall survival was 23 months. Patients with solitary bone metastasis had better overall survival than those with concomitant metastases (40 vs. 20 months; *p* < 0.001). In multivariate analysis, Memorial Sloan-Kettering Cancer Center risk group, en bloc resection, and synchronous solitary bone metastasis were predictors of better overall survival [56]. For patients with solitary bone metastasis without concomitant organ metastases at the initial diagnosis of renal cell carcinoma, resection of bone metastasis should be considered because local tumor control can be achieved and overall survival may be extended by resection.

**Fig. 3:**
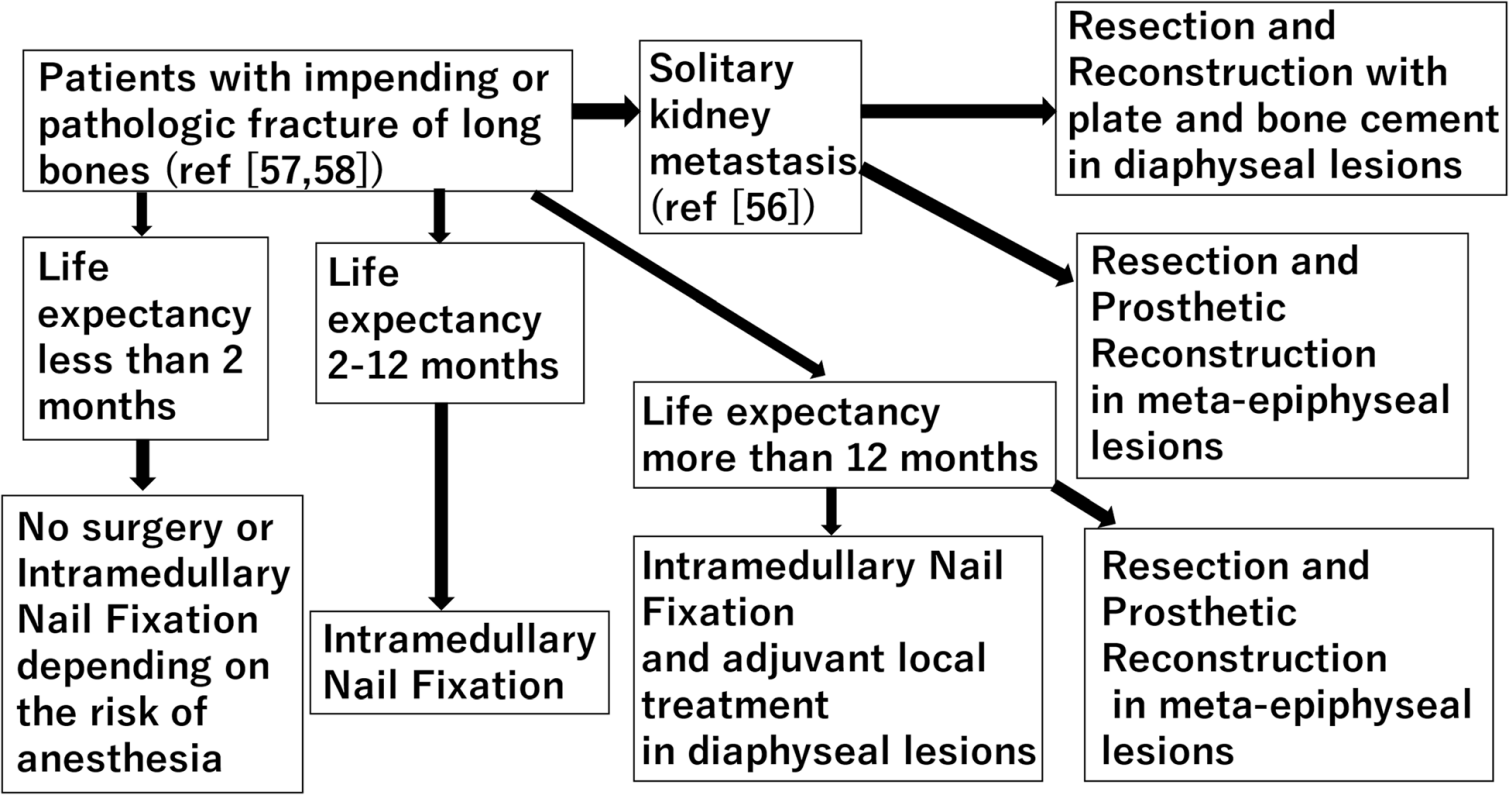
The treatment algorithm for bone metastases of the extremities

Most complications after intramedullary nail fixation occur one year after surgery; in contrast, prosthetic reconstruction seems to be a more durable surgical procedure but can cause complications earlier, within a year after surgery [57]. A recent study showed that pathological C-reactive protein and a tumor with an unfavorable diagnosis were poor prognostic factors for 1-year survival in patients undergoing surgery for long bone metastases. Based on these results, patients were divided into three different prognostic groups: (A) good prognosis primary tumor and physiological C-reactive protein with 1-year survival rate of 89 %; (B) poor prognosis primary tumor and physiological C-reactive protein or good prognosis primary tumor and pathological C-reactive protein with 1-year survival rate of 57 %; (C) poor prognosis primary tumor and pathological C-reactive protein with 1-year survival rate of 13 % [58]. Orthopedic surgeons may use these prognostic factors to determine how to operate on very fragile patients with bone metastases. Thio et al. created machine learning models to predict 90-day and 1-year survival rates in patients with bone metastases of the limb. The model is available on the following website: https://sorg-apps.shinyapps.io/extremitymetssurvival/ [59]. For patients with a short life expectancy (less than 12 months), less invasive surgery, such as intramedullary nail fixation, is indicated. In contrast, reconstruction of a more durable prosthesis is indicated for patients with a life expectancy of more than 12 months. Following these studies, we suggest a treatment algorithm for bone metastases of the extremities (Fig. 3) [56–58].

Philipp et al. found that patients with femoral metastases who underwent prophylactic internal fixation had a lower mortality rate than patients treated for pathologic femur fractures (*p* = 0.002) [60]. El Abiad et al. reinforced this data and reported that prophylactic fixation was related to a lower risk of complications (*p* = 0.02) and shorter hospital stay (*p* = 0.01) than post-fracture stabilization [61]. These studies suggest stabilizing an impending fracture because prophylactic fixation may increase survival and reduce complications, in addition to preventing a pathological fracture.

In summary of the  bone metastases section, the life expectancy of patients with metastatic bone disease appears to be the most important factor in determining the surgical methods that do not result in over- or under-treatment. Elevated C-reactive protein level was found to be an independent poor prognostic factor for long bone metastases at 1 year after surgery.

### Medical oncology

Due to the high recurrence rate and risk of complications after surgery, the development of new drugs for patients with symptomatic progressive TGCT is needed. Tap et al. evaluated the role of pexidartinib, a colony-stimulating factor 1 receptor inhibitor, in 120 patients with TGCT [62]. A higher proportion of patients in the pexidartinib group had tumor response compared to the placebo group (24 [39 %] of 61 vs. none of 59; *p* < 0.0001) [62]. Pexidartinib seems to be a promising systemic therapy for the treatment of TGCT in which surgery is not expected to improve the function of the affected limb.

A randomized, open-label phase II study comparing doxorubicin (75 mg/m^2^) and pazopanib (800 mg per day) in patients with metastatic soft tissue sarcoma over 60 years of age showed that progression-free survival in the pazopanib group was not inferior to that in the doxorubicin group, and grade 4 hematological toxicities occurred less frequently in the pazopanib group. The first-line treatment in patients over 60 years of age with metastatic soft tissue sarcoma is likely to be pazopanib [63].

A randomized, open-label phase III study that compared the efficacy of neoadjuvant chemotherapy between histology-tailored chemotherapy and standard anthracycline combined with ifosfamide chemotherapy in patients with localized high-risk soft tissue sarcoma showed that histology-tailored chemotherapy was not associated with better disease-free survival or overall survival. Therefore, the regimen of neoadjuvant chemotherapy for localized high-risk soft tissue sarcomas remains standard anthracycline plus ifosfamide chemotherapy [64].

A randomized, open-label phase III study that evaluated the efficacy of the addition of regional hyperthermia to neoadjuvant chemotherapy in patients with localized high-risk soft tissue sarcomas showed that the addition of local hyperthermia extended both local progression-free survival and overall survival. Therefore, regional hyperthermia should be added to neoadjuvant chemotherapy in patients with localized high-risk soft tissue sarcomas [65].

A single-arm, phase 2 study that evaluated the efficacy of pazopanib (800 mg per day) in patients with advanced chondrosarcoma showed that the disease control rate at 16 weeks was 43 % and the median progression-free survival was 7.9 months. Pazopanib seems to be effective in the treatment of chondrosarcoma [66].

A randomized study comparing the efficacy of consolidation treatment with high-dose chemotherapy (busulfan and melphalan) plus blood autologous stem cell rescue and standard chemotherapy (vincristine, dactinomycin, and ifosfamide, seven courses) in patients with localized Ewing sarcoma at high risk for relapse showed that high-dose chemotherapy improved event-free survival and overall survival [67].

It is important for the decision on the treatment plan for bone and soft tissue tumors, including bone metastases, to be made by a multidisciplinary team. If specialists are not in the same institute, videoconferencing or telemedicine is essential. In the coronavirus disease 2019 pandemic, videoconferencing has been mainly held to avoid close-contact settings, even when the specialists are in the same institute. Approximately 86 % of medical staff reported that all essential patient data were available for decision making and 89 % were satisfied with the time they spent discussing patients’ problems via videoconferencing [68]. Videoconferencing or telemedicine can expand the bone and soft tissue cancer care networks.

In summary of the medical oncology section, pexidartinib seems to be a promising systemic therapy in the treatment of TGCT for which surgery is unable to improve the function of the affected limb. The role of chemotherapy in the treatment of soft tissue sarcomas remains controversial.

### Future research

Multi-institute prospective comparative studies are required to answer priority research questions, such as the outcomes of orthopedic oncology reconstructions, clarify if less intensive surveillance of sarcoma patients affects survival, and the ideal surgical treatment (stabilization versus resection) in relation to the prognosis for metastatic cancer patients with impending or pathological fractures.

## Conclusions

With regard to primary bone tumors, computer navigation systems and 3D-printed prostheses seem to be new treatment options, especially in challenging anatomical locations, such as the sacrum and pelvis. Regarding the treatment of GCTB, recent studies have suggested that the administration of denosumab is related to a higher local recurrence rate following curettage but a lower local recurrence rate following en bloc resection. In addition, there was no difference in the local recurrence rate 5 years after surgery between short-term and long-term denosumab therapy. With regard to soft tissue tumors, percutaneous cryoablation appears to be a new treatment option for extra-abdominal desmoid tumors, with encouraging results. Regarding soft tissue sarcomas, a negative surgical margin of < 1 mm is sufficient to control local recurrence. Finally, the life expectancy of patients with metastatic bone disease appears to be the most important factor in determining the surgical method to be used. Elevated C-reactive protein level was found to be an independent poor prognostic factor at 1 year after surgery for long bone metastases.

## Data Availability

Not applicable.

## Abbreviations

3D: Three-dimensional
GCTB: Giant cell tumor of bone
TGCT: Tenosynovial giant cell tumor
ACT: Atypical cartilaginous tumor
MSTS: Musculoskeletal Tumor Society
MRI: Magnetic resonance imaging
TTI: Time to treatment initiation
NSAIDs: Non-steroidal anti-inflammatory drugs
PFS: Progression-free survival
PDGFR: Platelet-derived growth factor receptor
